# Comparative proteomics of three *Giardia lamblia* strains: investigation of antigenic variation in the post-genomic era

**DOI:** 10.1017/S0031182020000657

**Published:** 2020-08

**Authors:** Joachim Müller, Sophie Braga, Anne-Christine Uldry, Manfred Heller, Norbert Müller

**Affiliations:** 1 Vetsuisse Faculty, Institute of Parasitology, University of Bern, Länggass-Strasse 122, CH-3012 Bern, Switzerland; 2 Proteomics & Mass Spectrometry Core Facility, Department for BioMedical Research (DBMR), University of Bern, Freiburgstrasse 15, CH-3010 Bern, Switzerland

**Keywords:** Diversity, drug susceptibility, isolates, parasite–host interaction

## Abstract

*Giardia lamblia* is a causative agent of persistent diarrhoea widespread in regions with low hygienic standards. Laboratory research is based on cloned lines issuing from various patient isolates typed in the late 1980s and 90s using restriction analysis and serology. In the present study, we compared the well-characterized strain WBC6 with another clone of the parent WB isolate termed WBA1 and with a clone from another isolate, GS/M-83-H7, using shotgun mass spectrometry proteomics. We identified 398 proteins differentially expressed between the GS and both WB isolates and 97 proteins differentially expressed between the two WB isolates. We investigated the expression levels of the predominant variant-specific surface proteins (VSPs) in each clone and matched the previously described major VSPs of each strain to the corresponding open reading frame sequences identified by whole-genome sequencing efforts. Furthermore, since the original WB isolate comes from a patient treated with metronidazole, we compared the susceptibilities of the strains to nitro compounds, as well the expression levels of enzymes involved in nitro reduction and on the corresponding enzyme activities and found distinct differences between the three strains.

## Introduction

The diplomonadid *Giardia lamblia* (syn. *G. duodenalis*, *G. intestinalis*), an early diverging, anaerobic eukaryote (Adam, 2001; Cernikova *et al*., 2018), is a causative agent of persistent diarrhoea widespread in regions with low hygienic standards (Hemphill *et al*., 2019). After stomach passage, cysts ingested *via* contaminated food or water or *via* direct personal contact transform into trophozoites which colonize the duodenum and cause the symptoms peaking around 1 week post infection. In general, hosts in good physical condition recover within 2–3 weeks. In rare cases, the infection persists and becomes chronical causing severe damage of the intestinal epithelium (Allain *et al*., 2017) occasionally resulting in the development of irritable bowel syndrome (Litleskare *et al*., 2018). Eight different genotypes or assemblages, labelled A to H, have been identified, assemblages A and B having the broadest spectrum identifying animals as well as humans (Heyworth, 2016). Furthermore, assemblage E isolates may cause human giardiasis, as well (Zahedi *et al*., 2017). Thus, giardiasis occurs in humans as well as in other mammals and can be regarded as a zoonosis (Thompson, 2004). Human giardiasis is treated with the nitroimidazole metronidazole as first-line and the benzimidazole albendazole as second-line drug. Quinacrine, one of the first antigiardial drugs, is not in use, anymore (Gardner and Hill, 2001; Nash, 2001).

The largest part of laboratory research is based on the strain WBC6, an assemblage A1 strain obtained by limited dilution cloning from the WB isolate obtained from duodenal contents from a patient suffering from chronical giardiasis refractory to either quinacrine or metronidazole (MET) treatments (Smith *et al*., 1982*b*). The isolate originates from Afghanistan (Smith *et al*., 1982*a*) and was axenized at the NIH (Nash *et al*., 1985). The WBC6 strain, cloned by F. Gillin *via* limited dilution in 1983 (Campbell and Faubert, 1994), eagerly produces cysts *in vitro* (Campbell and Faubert, 1994), is amenable to genetic manipulation (Furfine and Wang, 1990; Sun *et al*., 1998), and its growth *in vitro* is less affected by the culture medium composition than other strains (own observations, see ‘Materials and methods’ section). Consequently, WBC6 is extensively used to investigate intracellular processes associated with en- and excystation of the parasite (Faso and Hehl, 2011; Zamponi *et al*., 2017) and has been the first *Giardia* strain where the complete genome has been sequenced (Morrison *et al*., 2007). Unfortunately, in many articles before this hallmark, the strain is referred to as WB only. This is insofar confusing, as other clones from the same original isolate with distinct characteristics exist. The most striking of these characteristics is related to a unique feature of *G. lamblia*, namely antigenic variation (Nash, 2002; Adam *et al*., 2010). The genome of WBC6 and of other strains that have been sequenced so far (see www.giardiadb.org) contain several hundred open reading frames (ORFs) encoding the so-called variant-specific surface proteins (VSPs). According to a recently published chromosome-scale reference genome, the genes encoding for VSPs, as well as other highly repetitive genes, are distributed over all five chromosomes (Xu *et al*., 2020). VSPs have the following properties in common: a signal peptide targeting the protein to the surface, a highly variable N-terminus with frequent CXXC-motifs (where X may be any amino acid), a nearly invariant C-terminus of 38 amino acids containing a CRGKA motif, a hydrophobic domain forming the membrane anchor (Adam *et al*., 2010). The N-terminus of VSPs is highly immunogenic. VSPs therefore constitute the main surface antigens of *G. lamblia* trophozoites. According to a generally admitted hypothesis, one single trophozoite expresses only one VSP at the same time (Nash *et al*., 1990*b*, 2001). During encystation, however, several VSPs may be expressed by the same cell (Carranza *et al*., 2002). The switching from one VSP to another, thus antigenic variation, at least indirectly depends in all likelihood, on posttranscriptional mechanisms (Kulakova *et al*., 2006; Prucca *et al*., 2008, 2011; Prucca and Lujan, 2009).

Studies in the 80s and 90s based on monoclonal antibodies raised against specific epitopes revealed the presence of major VSPs in a given WB trophozoite population. A well-characterized member of these major surface antigens is VSPA6 (in the literature also referred to as CRP170 due to approximate size of 170 kDa) that is expressed by several subclones of the WB isolate (Adam *et al*., 1988). VSPA6 is recognized by the cytotoxic monoclonal antibody (mAb) 6E7. Trophozoites resistant to mAb 6E7 express a different pattern of VSPs (Nash *et al*., 1988). Even in the absence of this selective pressure, other VSPs recognized by different monoclonal antibodies appear after many generations (Nash *et al*., 1990*a*).

According to further studies, mAb 6E7 recognizes an octapeptide motif (GAAPLYKK) present in 65 amino acid repeats distributed in different numbers over the VSP primary sequence (Mowatt *et al*., 1994) suggesting an evolution by gene duplication (Yang and Adam, 1995).

Conversely, trophozoites of WBC6 express a predominant VSP, named TSA417, exhibiting a peptide molecular mass of 72.5 kDa as predicted by the corresponding gene sequence (Gillin *et al*., 1990). Complement-dependent killing of trophozoites during exposure to anti-recombinant TSA417 antiserum provides a selective advantage for minor populations of non-TSA417-expressing trophozoites already present in the original culture (Meng *et al*., 1993). Furthermore, WBC6 trophozoites bearing non-TSA417-type VSPs appear during the process of excystation (Meng *et al*., 1993; Svärd *et al*., 1998).

Another extensively studied *G. lamblia* strain, namely clone GS/M-83-H7, representing an assemblage B genotype, originates from the human isolate GS obtained in Alaska and axenized by isolation of trophozoites from infected neonatal mice (Nash *et al*., 1985). This clone expresses a major surface antigen, VSP H7, which is immuno-reactive to a specific, cytotoxic mAb (mAb G10/4). VSP H7 has an apparent molecular mass of ca. 57 kDa (Nash and Mowatt, 1992). For GS/M-83-H7, *in vitro* antigenic variation replacing VSP H7 by diverse other VSPs on the surface of trophozoites occurs at about one variation event per 6.5 generations in comparison to one variation event per 12–13 generations in the original WB isolate (Nash *et al*., 1990*a*). Since GS/M-83-H7 infects humans as well as mice, immune responses to VSP H7 have been investigated not only *in vitro* (Müller *et al*., 1996) but also in mouse models (Gottstein *et al*., 1990; Müller and Gottstein, 1998). These studies show that antigenic variation occurs *in vivo* as a reaction to humoral immune responses in both hosts (Nash *et al*., 1990*c*; Bienz *et al*., 2001; Müller and von Allmen, 2005).

Recent shotgun mass spectrometry proteome studies have revealed that WBC6 trophozoite populations and trophozoite populations from other assemblage A strains express an impressive number of VSPs at the same time (Emery *et al*., 2014, 2015). Other studies have revealed that strain-dependent antigenic diversification of trophozoites occurs upon selective drug pressure (Emery *et al*., 2018; Emery-Corbin *et al*., 2018; Müller *et al*., 2019).

Using this sensitive method, we compared the well-characterized strain WBC6 with another clone of the parent WB isolate in the following termed WBA1. This clone grows only in a culture medium with strictly defined components and is refractory to antigenic variation, as previously shown (Nash *et al*., 1990*a*), and according to our own observations. Moreover, this strain is refractory to encystation (Campbell and Faubert, 1994). We included the strain GS/M-83-H7 as an outgroup into our comparison expecting that more proteins are differentially expressed between this clone and either WB clone than between both WB clones. We were particularly interested in the expression levels of the predominant VSPs in each clone and matched the previously described major VSPs of each strain to the corresponding ORF sequences identified by whole-genome sequencing efforts. Furthermore, since the original WB isolate comes from a patient treated with the nitroimidazole metronidazole, and since antigenic variation has been shown to be influenced by drug exposure (Müller *et al*., 2019), we compared the susceptibilities of the strains to nitro compounds and had a closer look on enzymes involved in nitro reduction and on the corresponding enzyme activities.

## Materials and methods

### Chemicals

If not otherwise stated, all biochemical reagents were from Sigma (St Louis, MO, USA). Nitazoxanide (NTZ) was synthesized at the Department of Chemistry and Biochemistry, University of Bern, Switzerland (Ch. Leumann). NTZ, albendazole (ALB) and MET were kept as 100 mm stock solutions in DMSO at −20°C.

### Axenic culture, harvest and storage of *G. lamblia* trophozoites

Trophozoites from *G. lamblia* WBC6 (C6), WBA1 (A1) and GS/M-83-H7 (H7) were grown under anaerobic conditions in 10 mL culture tubes (Nunc, Roskilde, Denmark) on modified TYI-S-33 medium as previously described (Clark and Diamond, 2002). In order to ensure the growth of the A1 and H7 clones, the components were as close as possible to the isolation medium, in particular heat-inactivated adult bovine serum (Biofluids, Rockville, MD, USA) and casein peptone (Becton Dickinson, Cockeysville, MD, USA). Prior to shotgun mass spectrometry analysis, the cultures were routinely passaged two times. Subcultures were performed by inoculating 100 *μ*L of cells from a confluent culture detached by cooling (see below) to a new tube containing 10 mL culture medium (Müller *et al*., 2006). Trophozoites were harvested by incubation on ice for 15 min followed by centrifugation (300 × g, 10 min, 4°C). Pellets were washed three times with ice-cold PBS, counted and stored at −80°C for subsequent proteomic analysis or for enzymatic assays.

### Proteomics

Cell pellets were lysed in 100 *μ*L 8 m urea/100 mm Tris/HCl pH 8/cOmplete^TM^ protease inhibitor cocktail (Roche Diagnostics, Rotkreuz, Switzerland) by incubation for 15 min at room temperature followed by 15 min in an ultrasonic water bath. Proteins were reduced and alkylated with 10 mm DTT for 30 min at 37°C and 50 mm iodoacetamide for 30 min at 37°C. Proteins were precipitated at −20°C by addition of 5 volumes cold acetone and incubation at −20°C overnight. All liquid was carefully removed and the pellet dried in ambient air for 15 min before reconstitution of proteins in 200 *μ*L of 8 m urea, 50 mm Tris-HCl pH 8.0. Protein concentration was determined by Bradford assay and an aliquot corresponding to 10 *μ*g protein was digested by trypsin (1:50 trypsin/protein ratio) for 6 h at 37°C after dilution of urea concentration to 1.6 m with 20 mm Tris-HCl pH 8.0 and 2 mm CaCl2. The digests were acidified with TFA (1%) and analysed by LC-MS/MS. Three repetitive injections of an aliquot corresponding to 500 ng protein digest was separated on an EASY-nLC 1000 coupled to a QExactive mass spectrometer (ThermoFisher Scientific). Peptides were trapped on an Acclaim PepMap100 C18 pre-column (3 *μ*m, 100 A˚, 75 *μ*m × 2 cm, ThermoFisher Scientific, Reinach, Switzerland) and separated by backflush on a C18 column (3 *μ*m, 100 A˚, 75 *μ*m × 15 cm, Nikkyo Technos, Tokyo, Japan) by applying a 60 min gradient of 5% acetonitrile to 40% in water, 0.1% formic acid, at a flow rate of 400 nL min^−1^. Peptides of m/z 360–1400 were detected with a resolution of 70 000 applying an automatic gain control (AGC) target of 1E06 and a maximum ion injection time of 50 ms. A top 10 data-dependent method for precursor ion fragmentation with a stepped 27% normalized collision energy was applied with the following settings: precursor isolation width of 2 m/z, resolution 17 500, AGC of 1E05 with a minimum target of 1E03, maximum ion time of 110 ms, charge exclusion of unassigned and 1+ ions, peptide match on and dynamic exclusion for 20 s, respectively.

The MS data were obtained from three biological replicates, with three technical replicates for each biological replicate, for each strain. All MS data were processed by MaxQuant (version 1.5.4.1) with matching between runs for the same strain activated, but not between different strains, in order to avoid over-interpretation of the data. The sample sets were interpreted separately by MaxQuant. Fragment spectra were interpreted against a recent *Giardia* protein sequence database including both WB and GS datasets in fasta format (GiardiaDB-5.0_GintestinalisAssemblageA_AnnotatedProteins; GiardiaDB-5.0_GintestinalisAssemblageB_AnnotatedProteins_v2). The trypsin cleavage rule allowed amide bond cleavage after lysine and arginine but not if a proline follows and up to three missed cleavage sites, fixed carbamidomethylation modification of cysteines, variable oxidation of methionine and acetylation of protein N-termini. Precursor and fragment mass tolerances were set to 10 and 20 ppm, respectively. Peptide spectrum matches, peptide and protein group identifications were filtered to a 1% false discovery rate (FDR) based on reversed database sequence matches, and a minimum of two razor or unique peptides were required to accept a protein group identification. Protein identifications considered as contaminations (e.g. trypsin or BSA) as well as proteins identified only by site (considered by MaxQuant developers as very likely false positives) were removed for statistical validation. The normalized label-free quantification (LFQ) protein group intensities as calculated by MaxQuant were used for relative proteome quantifications. First, we imputed missing protein LFQ values for samples in any condition group when there were at least two LFQ intensities in one group (downshift of 2.5 s.d. with a width of 0.3 s.d.). When comparing VSPs only, peptides unique to a single VSP protein were used for the calculation of protein intensities based on the sum of the three most intense peptides (Top3 approach). Before summing, missing peptide intensities were imputed sample group wise, when there were at least two valid intensities (downshift of 1.8 s.d. with a width of 0.3 s.d.). The resulting protein intensities were named iTop3. This process left proteins without values in one or the other group. For Welch's *t*-tests, those missing protein intensities were replaced by imputed values from the very low end of intensity distributions in analogy to the LFQ imputation (downshift 2.5 s.d., width of 0.3 s.d.). An FDR-controlled Benjamini–Hochberg procedure was used for the correction of *P* values. A log2-fold change of at least one and a corrected *P* value of 0.05 were required to be considered as significant. Statistical testing and imputation were made with a home-made R script run under R-Studio. Our main goal was to identify the VSPs in the H7 proteome. To do this, we had to use the assemblage B database. Since most of the other proteins share high homologies with those from assemblage A strains and are found in both databases, they are called orthologues. We eliminated those proteins that were expressed in both strains sharing the same ORF numbers (e.g. GL50803_10167 in assemblage A and GSB_10167 in assemblage B) *via* the GenesbyOrthologues tool on the GiardiaDB site (www.giardiabd.org). Small differences in peptide sequences or SNPs were not considered. They can easily be identified by comparing the sequences of orthologues provided by GiardiaDB. For comparative quantification of orthologues, the LFQ values corresponding to GL50803 were used for assemblage A strains and the values corresponding to GSB were used for assemblage B strains.

The mass spectrometry proteomics data have been deposited to the ProteomeXchange Consortium *via* the PRIDE (Perez-Riverol *et al*., 2019) partner repository with the dataset identifier PXD017597.

### Drug susceptibility assays

Drug susceptibility of *G. lamblia* trophozoites of the strains C6, A1 and H7 was tested in 96-well plates inoculated with 10^3^ trophozoites per well and MET, NTZ or ALB at various concentrations (dilution factor 2) and incubated in an anaerobic growth chamber (85% N_2_, 10% H_2_, 5% CO_2_). After 72 h (C6, A1) or 96 h (H7), growth of cells was monitored by a vitality assay based on the reduction of resazurin (Alamar Blue) to a pink product that was assayed fluorimetrically (Bénéré *et al*., 2007). The IC_50_ values were determined using the logit-log algorithm as described (Müller and Hemphill, 2013).

To determine minimal inhibitory concentrations (MIC), WT and C4 trophozoites were inoculated in the presence of increasing amounts (dilution series by a factor 2) of the nitro compounds MET, NTZ or ALB as a control. The tubes were incubated at 37°C for 96 h (C6, A1) or 120 h (H7). The MIC was determined by observing the wells under the microscope starting from higher to lower concentrations. The concentration at which the first living trophozoites were visible is given as the MIC (Müller *et al*., 2018).

### Enzyme assays

Trophozoite crude extracts were prepared by suspending trophozoite pellets in 50 mm Tris-Cl^−^, pH 7.0, containing 0.05% triton-X-100 and a protease inhibitor mix (Halt, ThermoFisher Scientific, Waltham, MA) according to the manufacturer's instructions. NAD(P)H oxidase and quinone reductase activities were measured in 96-well microtiter plates containing 100 *μ*L of a reaction mix containing buffer (50 mm Tris-Cl^−^, pH 7.0), 0.5 mm thiazolyl blue tetrazolium (MTT), 0.5 mm NADH or NADPH and 5 *μ*L of crude extract. To measure quinone reductase activity, 0.1 mm menadione was added to the mix. The plates were incubated at 37°C under aerobic conditions. Substrate and enzyme blanks were included. After different time points, the reaction was stopped by adding 100 *μ*L of pure ethanol thus solubilizing the product formed by the reduction of MTT, formazan (Prochaska and Santamaria, 1988; Müller *et al*., 2015). Nitroreductase activity was quantified *via* the reduction of 7-nitrocoumarin (7-NC) to 7-aminocoumarin (7-AC) using the reaction mix as described above, without MTT and 0.1 mm 7-NC, with the same volumes and under the same conditions of incubation. Enzyme and substrate blanks were included. The reaction was stopped by adding 100 *μ*L of 50 mm HCl lowering the pH to allow full protonation of the reaction product (Wagner, 2009). 7-AC was quantified by fluorimetry with excitation at 365 nm and emission at 455 nm (Müller *et al*., 2015). Absorption at 590 nm (MTT-based assays) and fluorescence intensities were quantified using a 96-well multimode plate reader (Enspire; Perkin-Elmer, Waltham, MA, USA). Protein contents of crude extracts were determined by the Bradford method (Bradford, 1976) using a commercial kit (Biorad, Hercules, CA, USA).

## Results and discussion

### Mass spectrometry analysis of proteins expressed in *G. lamblia* trophozoites

**Fig. 1.**
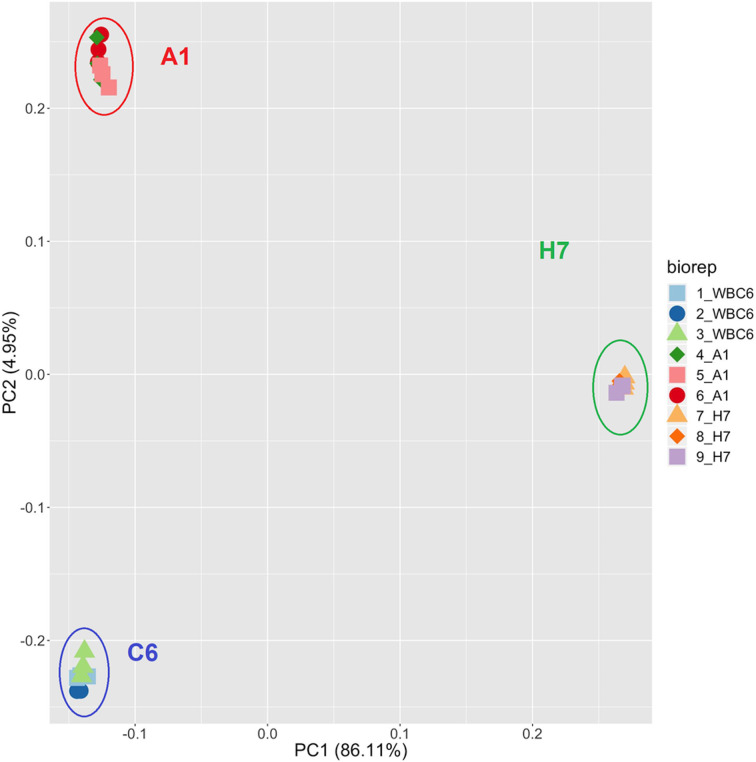
Principal component analysis of proteome dataset from *G. lamblia* trophozoites of different strains. Trophozoites of the strains WBC6 (C6), WBA1 (A1) and GS/M-83-H7 (H7) were compared by MS shotgun analysis as described in ‘Materials and methods’ section. For each strain, all technical and biological (square, circle, diamond) replicates are shown.

Shotgun mass spectrometry of the proteomes of trophozoites of the strains WBC6 (C6), WBA1 (A1) and GS/M-83-H7 (H7) allowed the identification of 24 739 unique peptides matching to 2 368 proteins (Table S1). Overall analysis of the data by principal component analysis (PCA) revealed that biological and technical replicates clustered together for each strain. The proteomes of the three strains formed non-overlapping clusters. A1 and C6 were separated by PC2 only, whereas the proteome of H7 was separated by both other strains PC1 and PC2 (Fig. 1). A PCA with strains A1 and C6 only is shown in Fig. S1.

### Differentially expressed proteins

**Fig. 2.**
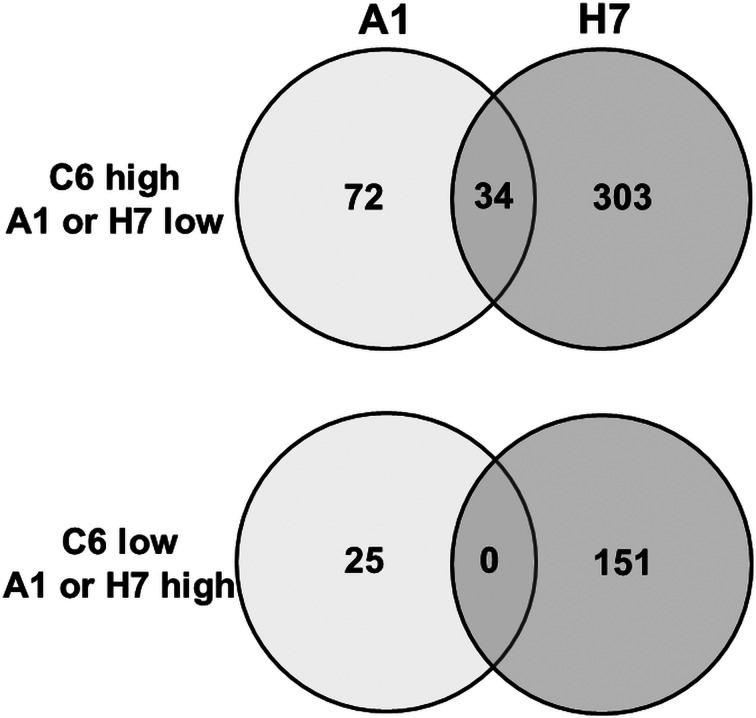
Venn diagram detailing the number of differentially expressed proteins in *G. lamblia* trophozoites of different strains. Trophozoites of the strains WBC6 (C6), WBA1 (A1) and GS/M-83-H7 (H7) were subjected to MS shotgun analysis as described in ‘Materials and methods’ section. Orthologues present in both A and B strains have been subtracted using the GenesbyOrthologues function implemented in the GiardiaDB.

To get a better estimation of ‘true’ differentials between assemblage and B strains, orthologues present in both A and B strains have been subtracted using the GenesbyOrthologues function implemented in the GiardiaDB. Without eliminating these orthologues from the comparison, WBC6 *vs* H7 would yield 1320 differentials, WBA1 *vs* H7 would yield 1264 differentials. This analysis revealed that between strains C6 and A1, 97 proteins were differentially expressed, as compared to 454 proteins between strains C6 and H7 (Fig. 2). Comparison of differentially expressed proteins between the strains H7 *vs* the strains C6 and A1 revealed that 253 proteins had higher levels in both WB strains than in H7, and 146 proteins had higher levels in H7 than in the WB strains. Besides hypothetical proteins, the categories with the highest numbers of differentials comprised proteins involved in cytoskeleton, adhesion and organelle transport, intermediary metabolism, gene expression and development, and signalling (Table 1). Strain H7 expressed a unique set of 13 VSPs different from the VSP sets expressed in both WB strains. H7 and the WB clones belong to different assemblages, it is not surprising that an order of magnitude more differential proteins are identified between H7 and the WB clones than between the two WB clones. Some of the proteins overexpressed in H7, in particular proteins involved in signalling or attachment, may contribute to the broad host spectrum of this strain, which infects humans as well as animals, as reviewed elsewhere (Müller and von Allmen, 2005).

**Table 1.** Number of differentially expressed proteins in strain GS/M-83-H7 as compared to both WBC6 and WBA1 trophozoites

(Hypothetical) function	Higher levels in WBC6 and WBA1	Higher levels in GS/M-83-H7
Variant-specific surface proteins	4	13
Cytoskeleton, adhesion and organelle transport	34	15
Intermediary metabolism	54	22
Signalling	16	13
Gene expression, cell cycle, development	48	17
Chaperones	10	0
Transport of metabolites	9	5
Hypothetical	82	60
Total	253	145

The strains were grown and processed for MS shotgun analysis as described in ‘Materials and methods’ section. The proteins are grouped with respect to their hypothetical functions.

When comparing the set of the 97 differentially expressed proteins between C6 and A1 in more detail, it turned out that – besides 32 hypothetical proteins – 22 VSPs differed between these strains. Eleven differential proteins were involved in signalling, 11 cytoskeleton-related proteins, 10 involved in intermediary metabolism, 10 proteins with functions in gene expression and development, and one with a chaperone-like function (Table 2). Thirty-four proteins were also significantly lower in strain H7 as compared to C6 (Fig. 2), in particular the 15 C6-specific VSPs (see ‘Antigenic variation’ section) and 19 proteins with various functions, as highlighted in Table 2.

**Table 2.** Overview of ORFs differentially expressed in WBC6 (C6) as compared to WBA1 (A1) trophozoites

(Hypothetical) function	Protein	ORF number in *Giardia* database
Higher expression in C6		
Variant-specific surface proteins	*VSP-88*	101 074
	*VSP-169*	112 801
	*VSP-44*	113 450
	*VSP-5*	113 797
	*VSP-149*	116 477
	*VSP-38*	13 194
	*VSP-160*	137 612
	*VSP-188*	137 613
	*VSP-77*	137 617
	*VSP-8*	137 618
	*VSP-3*	137 740
	*VSP-186*	14 586
	*VSP-100*	33 279
	*VSP-49*	41 472
	*VSP-121*	5812
Cytoskeleton, adhesion and organelle transport	Protein 21.1	101 168
	Ankyrin repeat protein	14 745
	IFT complex B	17 223
	Protein 21.1 (Ankyrin repeat)	17 608
	*ENC6 protein*	24 372
	Trichohyalin	32 375
	Protein 21.1	3760
	*Cyst wall protein 2*	5435
	*Synaptic glycoprotein SC2*	88 581
	Dynein heavy chain	94 440
Intermediary metabolism	*Ubiquitin carboxyl-terminal hydrolase 14*	102 710
	Peroxiredoxin 1	14 521
	2,5-diketo-D-gluconic acid reductase	151 884
	NAD(P)H:menadione oxidoreductase	17 150
	Nitroreductase Fd-NR2	22 677
	L-serine dehydratase	24 662
	Glucosamine-6-phosphate deaminase	8245
	*Adenylate kinase*	90 402
Signalling	Kinase, AGC PKA	11 214
	Kinase, NEK	13 215
	Kinase, NEK	137 733
	Kinase, CAMK CAMKL	14 661
	*ARF GAP*	17 561
	AMP-activated protein kinase, gamma-1 subunit	3414
Gene expression, cell cycle, development	Chromosome segregation protein SMC	10 027
	Threonyl and alanyl tRNA synthetase second additional domain	150 018
	CDC19	17 413
	Golgi/cell cycle associated protein, putative	17 472
	Preimplantation protein 3	3417
	SBDS C-terminal domain protein, putative	40 521
	*Pelo protein*	5892
Chaperones	T-complex protein 1, *β* subunit	11 397
Hypothetical		(25 proteins)
		*(12 proteins also in H7)*
Higher expression in A1		
Variant-specific surface proteins	High cysteine membrane protein VSP-like	112 135
	High cysteine membrane protein Group 1	11 309
	VSP-175	137 608
	High cysteine membrane protein Group 1	16 318
	CXC-rich protein	17 476
	VSP A6 (annotated as hypothetical protein)	221 693
	High cysteine membrane protein Group 6	41 942
Cytoskeleton, adhesion and organelle transport	Dynein heavy chain	17 243
Intermediary metabolism	Glycosaminoglycan polysaccharide lyase	10 238
	Nitroreductase family protein	15 307
Signalling	Kinase, NEK	14 216
	Kinase, CAMK CAMKL	17 566
	Serine/threonine protein phosphatase 5	21 498
	Protein kinase NEK family protein	152 721
Gene expression, cell cycle, development	MCM2	15 344
	DNA replication licensing factor MCM6	17 390
	RNA binding protein, putative	92 031
Hypothetical		(8 proteins)

The strains were grown and processed for MS shotgun analysis as described in ‘Materials and methods’ section. For each strain, three biological replicates have been tested (with three technical replicates per biological replicate). The proteins in this list had significantly different expression levels both in iLFQ and iTop3 values. The proteins with different expression levels also in strain GS/M-83-H7 are printed in italics.

### Antigenic variation

The strains investigated in this study had been characterized with respect to their (major) surface antigens. Therefore, in a first step, we matched the original antigens by protein–protein blasts to sequences found in the GiardiaDB. It turned out that sequences attributed to TSA417 (strain C6) matched to GL50803_113797, annotated as VSP5 and to GL50803_113450, annotated as VSP44. Concerning strain H7, the sequence published for VSPH7 matched to GSB150963.

Surprisingly, the sequence of CRP170, the predominant surface antigen of strain A1, matched to a sequence annotated as a hypothetical protein, namely GL50803_221693 with 100% identity and a total score of 3491 (Fig. S2). The GL50803_221693 ORF encodes a 228 kDa protein containing 2259 amino acids. The identified peptides of GL50803_221693 covered unique protein sequences near the N terminus (aa 75–151), followed by 26 repeats of the peptide TNPSDPTGTCVSAVDCQGSAGYYTDDS-VSDAKECKKCNAPCTACAGTADKCTKCDANGAAPYLKK (thus containing the GAAPYLKK motif recognized by mAb6E7), and a unique sequence near the C-terminus (aa 1812–2223). Regions where trypsin produces too long/hydrophobic or too short peptides, namely the two transmembrane helices at the C- and N-terminal part of the protein, were missed as can be expected by the used technology (Fig. S3). Proteins encoded by other ORFs sharing high score homologies with CRP170, namely GL50803_137752 and GL50803_37093 (Fig. S2), were not detected in the present dataset.

**Fig. 3.**
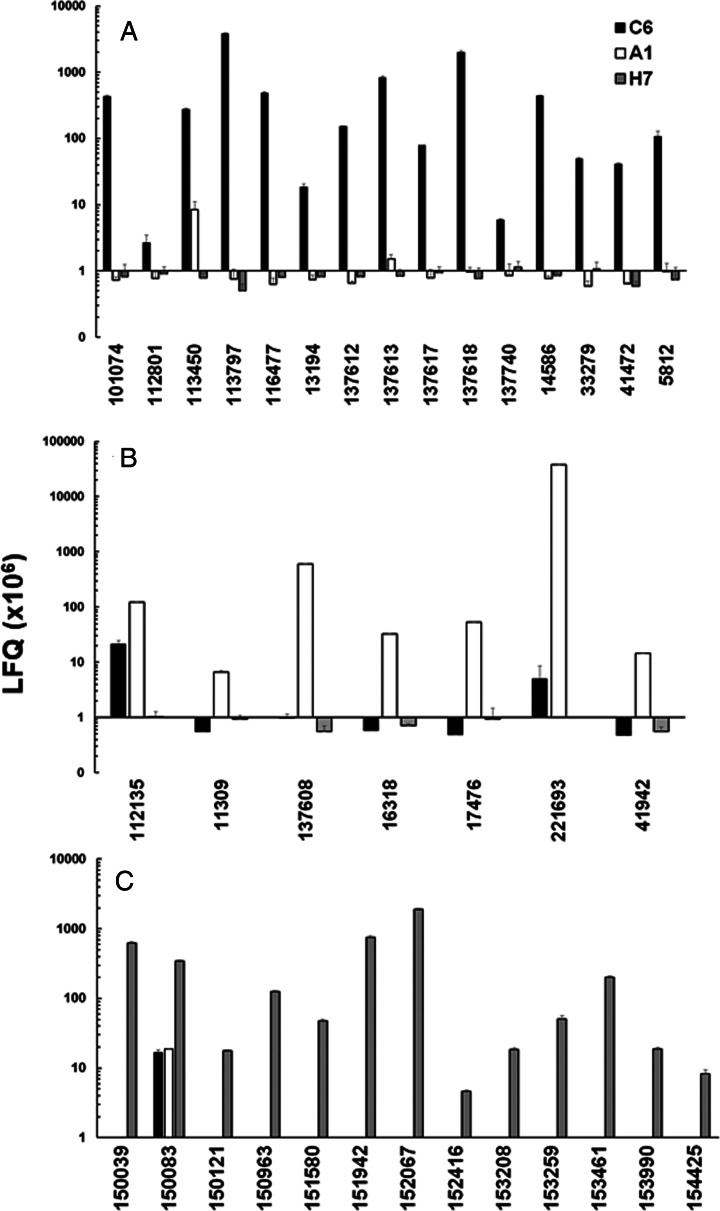
Quantitative assessments of the major variant-specific surface proteins (VSPs). Trophozoites of the strains WBC6 (C6), WBA1 (A1) and GS/M-83-H7 (H7) were subjected to MS shotgun analysis as described in ‘Materials and methods’ section. For all VSPs, mean values ± one standard deviation for LFQ intensities (×10^6^) in three biological replicates are shown. The VSPs are termed by their respective accession numbers in the GiardiaDB (GL50803 for C6 and A1; GSB for H7). A, VSP pattern of strain C6; B, VSP pattern of strain A1; C, VSP pattern of strain H7.

In a second step, we compared the LFQ intensities of the major VSPs in all strains (Fig. 3). In the case of strain C6, TSA417 (VSP5; ORF113797) was still one of the dominating VSPs, followed by VSP-8 (ORF 137618), VSP-188 (ORF 137613) and four other VSPs in the range of 10^7^ LFQ units (Fig. 3A). Amongst these VSPs were the gene products of ORFs 101074 and 137618. ORF 101074 (VSP88) was identical to the sequence of AAG16629, annotated as VSP9B10A (Nash *et al*., 2001). ORF 137618 (VSP8) covered AF293416, annotated as VSPB10B (Carranza *et al*., 2002) only to 88% leaving 70 amino acid gaps. In a previously published comparative proteomics study (Emery *et al*., 2015), a strain WB expressed four of the major VSPs identified in WBC6, namely VSP38 (ORF 13194), VSP49 (ORF 41472), VSP149 (ORF 116477) and the previously mentioned VSP188.

In strain A1, GL50803_221693 (homologous to VSPA6) was – by far – the most predominant VSP with LFQ values above 10^11^, but not the only one identified. The five next abundant VSPs had, however, expression levels three or more magnitudes lower than VSPA6. Interestingly, VSPA6 was expressed in strain C6 as well, but only at LFQ levels below 10^7^ (Fig. 3B). In strain H7, 13 VSPs with LFQ levels around 10^7^ or higher were identified. VSPH7 (GSB150963) was not predominant, five other VSPs having equal or higher expression levels (Fig. 3C).

Moreover, we have reinvestigated the most predominant VSPs of the three strains using only peptides labelled as being unique to a single VSP by using iTop3 protein intensities (see ‘Materials and methods’ section and Table S2). The corresponding values are shown as Supplementary Fig. S4. Some minor VSPs disappeared, but the overall pattern of the strains did not change as compared to the analysis based on LFQ values.

These results show that A1 had the most ‘homogeneous’ population of trophozoites with respect to their surface proteome. The two other strains yielded mixed trophozoite populations with five or more equally dominant subpopulations.

The fact that VSPA6 was expressed at low levels in strain C6 prompted us to investigate whether this VSP was found in the proteome of the same strain grown on a different medium. The corresponding dataset had been published earlier in a different context (Müller *et al*., 2019). Since the corresponding ORF (221693) was annotated as a hypothetical protein and not as a VSP, the corresponding expression levels had been overlooked. As shown in Table S3, VSPA6 became the third most abundant VSP in C6 trophozoites grown on a new culture medium differing from the old one merely with respect to the quality of the serum and the peptone. Moreover, VSPA6 had very high expression levels in two Australian assemblage A1 isolates investigated in the same study (Müller *et al*., 2019). Thus, VSPA6 cannot be regarded as a unique marker for WBA1 and related clones.

### Susceptibility to antigiardial drugs and reductase activities

**Fig. 4.**
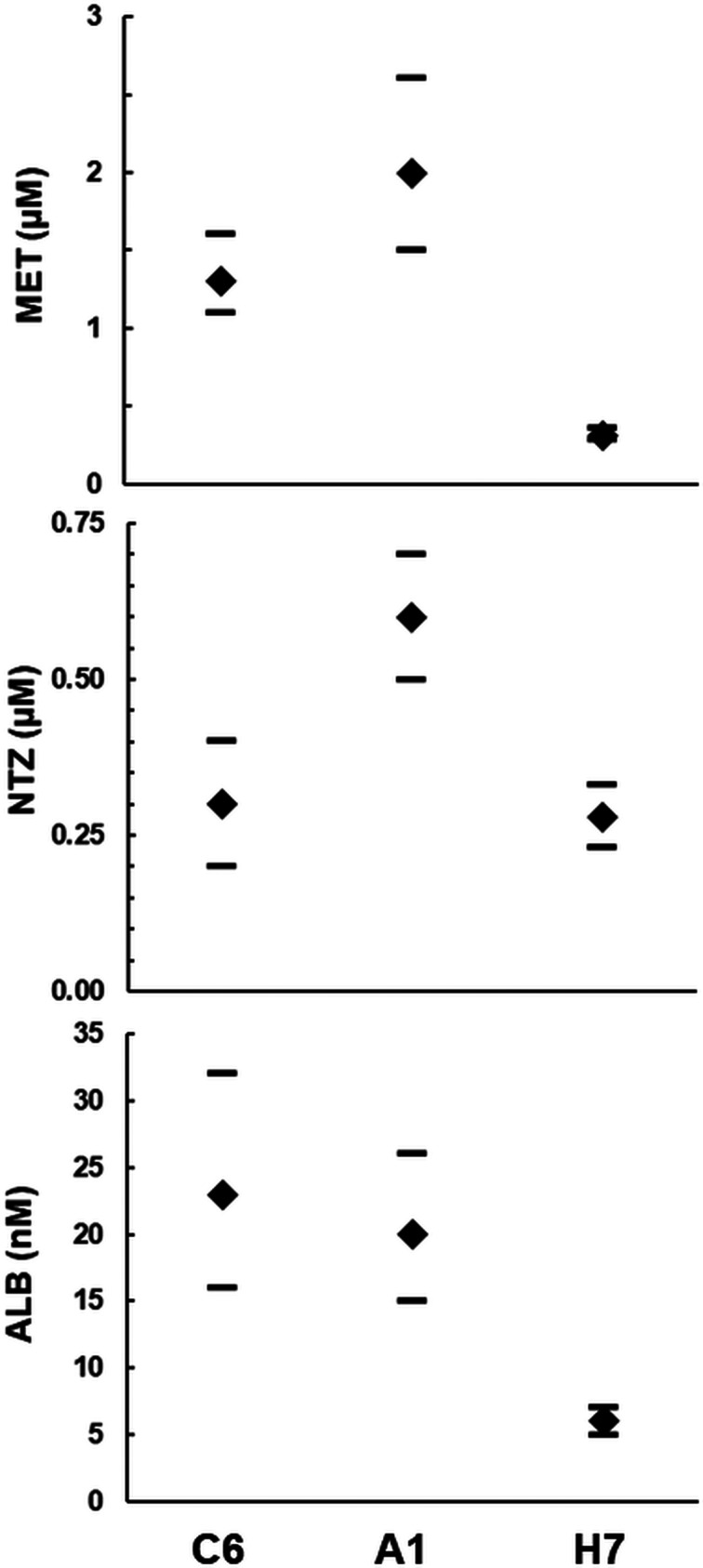
Determination of drug susceptibilities. The strains WBC6 (C6), WBA1 (A1) and GS/M-83-H7 (H7) were exposed to serial dilutions of the nitro compounds metronidazole (MET) and nitazoxanide (NTZ), as well as of albendazole (ALB). IC_50_ values were determined as described in ‘Materials and methods’ section and are given in *μ*m (MET, NTZ) or nm (ALB). Diamonds correspond to the IC_50_s, bars to the 95% confidence intervals.

Since the strains C6 and A1 had been isolated from a patient treated with MET, we were curious to investigate whether these strains differed in their susceptibilities to nitro drugs from the strain H7 isolated from a different source. Interestingly, strain A1 had higher IC_50_s than strain C6 when exposed to MET and to NTZ, but not to ALB. Strain H7 was more susceptible to MET and to ALB than both strains C6 and A1 (Fig. 4). The MIC of both nitro drugs were even nearly one order of magnitude higher on A1 than on both other strains (Table 3). When comparing IC_50_s and MICs from different strains, one should consider that these constants measure different things, as reviewed elsewhere (Müller and Hemphill, 2013). In the case of bacteria exposed to antibiotics, elevated MICs are regarded as indicative for ‘resistance’, whereas elevated IC_50_s without elevated MICs indicate ‘tolerance’ (Brauner *et al*., 2016). In our case, strain A1 would be at the edge of ‘resistance’ with, however, much lower MICs than those of resistant strains generated in the laboratory (Müller *et al*., 2018).

**Table 3.** Determination of minimal inhibitory concentrations (MICs)

Compound	C6	A1	H7
MET	6.25	50	6.25
NTZ	1.25	10	1.25
ALB	0.125	0.125	0.125

The strains WBC6 (C6), WBA1 (A1) and GS/M-83-H7 (H7) were exposed to serial dilutions of the nitro compounds metronidazole (MET) and nitazoxanide (NTZ), as well as of albendazole (ALB). MICs were determined as described in ‘Materials and methods’ section and are given in *μ*m.

**Fig. 5.**
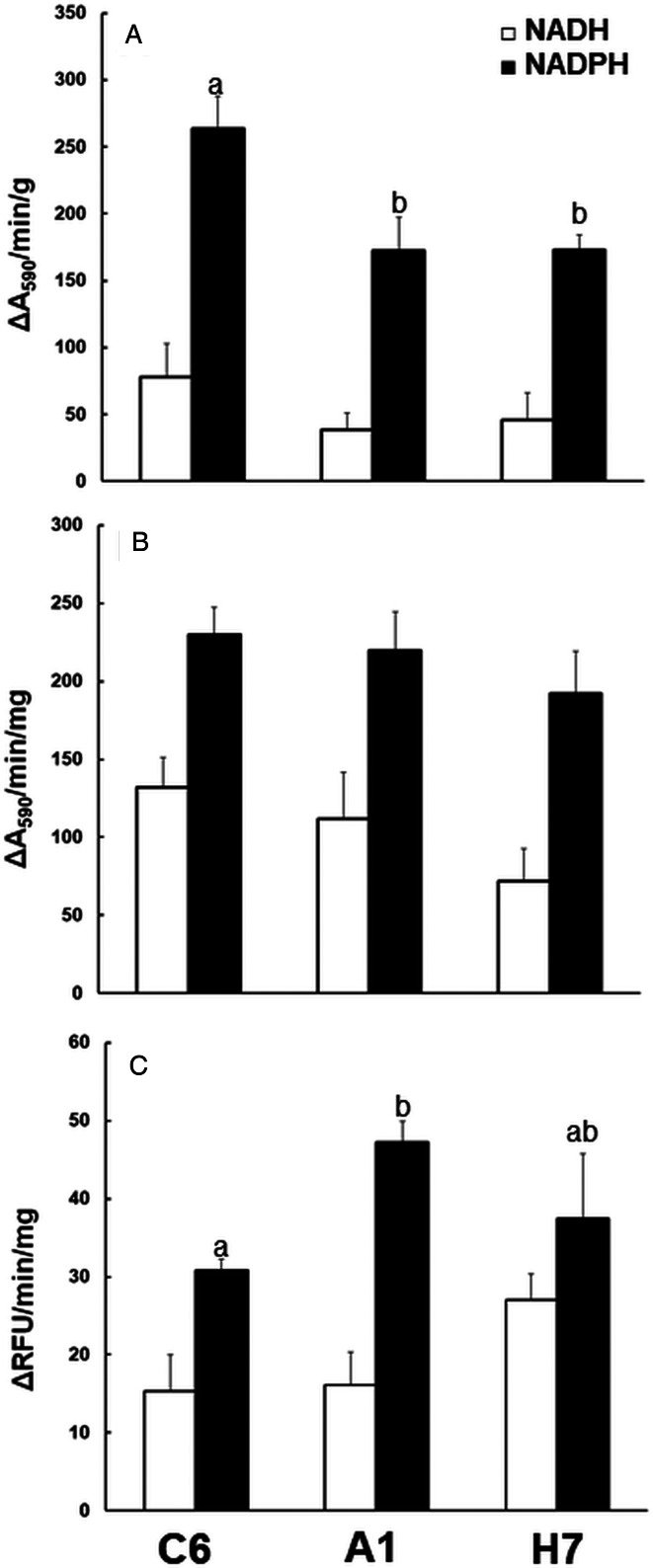
Reductase activities in crude extracts of *G. lamblia* trophozoites. The assays were performed as described in ‘Materials and methods’ section using either NADH (white bars) or NADPH (black bars) as cofactors. A, NAD(P)H oxidase; B, quinone reductase with menadione as a substrate; C, nitroreductase with 7-nitrocoumarin as a substrate. Mean values ± one standard deviation for three independent assays are given. Values superscribed by different letters are significantly different (two-sided *t*-tests; *P* < 0.05).

To see whether these differences matched to differences in reductase activities, we assayed NAD(P)H oxidase activity, quinone reductase activity and nitroreductase activity in crude extracts from the three strains (Fig. 5). Crude extracts from strains A1 and H7 had significantly lower NADPH oxidase, but not lower NADH oxidase activities than strain C6 (Fig. 5A). Quinone reductase activity with menadione as a substrate was at equal levels in crude extracts from all three strains (Fig. 5B). Nitroreductase activity, assayed as the full reduction of 7-NC to 7-AC was slightly, but significantly increased in A1 as compared to C6 crude extracts. Crude extracts from strain H7 had an intermediate activity (Fig. 5C).

### Enzymes involved in nitro reduction or detoxification processes

These findings prompted us to have a closer look at the expression levels of enzymes involved in nitro reduction and related detoxification processes. The values are listed in Table 4. Strain C6 had significantly higher levels of the nitroreductase NR1 than the two other strains. Conversely, A1 was the only strain expressing the homologous NR family protein without ferredoxin domain at its N-terminus. C6 and A1, but not H7, expressed a 2Fe-2S ferredoxin, the only free ferredoxin detected in the proteome of the strains investigated in this study. Moreover, the ferredoxin-domain protein hydrogenase 1 was identified in all strains with significantly higher levels in strain H7.

**Table 4.** Overview of proteins involved in reduction (and thus activation) of nitro compounds and the scavenging of radicals in trophozoites of the strains WBC6 (C6), WBA1 (A1) and GS/M-83-H7 (H7)

Annotation	DB	C6	A1	H7
Fd-NR2 (‘NR1’)	22 677	106 ± 5*	61 ± 1	62 ± 2
Fd 2Fe_2S	27 266	9 ± 1	10 ± 0	nd*
Fd-hydrogenase 1	6304	46 ± 1	45 ± 0	122 ± 9*
NR family (‘NR3’)	15 307	nd	4 ± 0*	nd
PFOR 1	17 063	1713 ± 22	1510 ± 29	2290 ± 20
PFOR 2	114 609	4619 ± 48	3966 ± 37	3938 ± 51
Thioredoxin-reductase	9827	243 ± 26	208 ± 20	226 ± 7
A-type flavoprotein	10 358	681 ± 11*	471 ± 10	461 ± 13
NAD(P)H oxidase lateral transfer	33 769	2324 ± 56	2103 ± 55	2032 ± 50
NAD(P)H oxidase	9719	490 ± 16*	332 ± 11	290 ± 3

Cells were harvested and subjected to MS shotgun analysis as described in ‘Materials and methods’ section. For each strain, three biological replicates have been tested (with three technical replicates per biological replicate). For all proteins, mean values ± standard errors for LFQ intensities (×10^6^) in three biological replicates are given (nd, below detection limit). DB, number in GiardiaDB; Fd, ferredoxin; LT, lateral transfer; NR, nitroreductase; PFOR, pyruvate-ferredoxin oxidoreductase. *, value is statistically significant from the two others (one-factorial ANOVA, *P* < 0.01).

C6 had significantly higher levels of A-type flavoprotein and of NAD(P)H oxidase than the two other strains. All other proteins investigated within this context, in particular both pyruvate-ferredoxin-oxidoreductases, did not differ between the strains (Table 4).

## Conclusions

Our results, in particular those concerning the surface proteome of the investigated strains, show that care must be taken when referring to these strains using the VSP terminology established three decades ago as a result of their reactivity with monoclonal antibodies. A given antibody may cross-react with epitopes on VSPs encoded by a different ORF with high sequence identity and/or with VSPs carrying epitopes with a similar secondary structure, as illustrated by the following example. Using the monoclonal antibody Mab9B10, for instance, two different VSPs, namely VSP9B10A (Nash *et al*., 2001) and VSP9B10B (Carranza *et al*., 2002) were identified, which correspond to two major VSPs (VSP88 for sure and VSP8 most likely) of strain WBC6 as shown in our dataset.

Consequently, the hypothesis that one single trophozoite expresses one single VSP could be modified in the sense that one single trophozoite may express VSPs that may be encoded by one or more ORFs. This hypothesis could be verified only by single-cell proteomics (Dou *et al*., 2019; Liu *et al*., 2020), a method which has not been established for *G. lamblia*, so far.

Rich in potential trypsin and chymotrypsin cleavage sites inside a highly repetitive motif (covering three-quarters of its primary sequence in the case of VSPA6), the digest by pancreas proteases (or by proteases released by decaying trophozoites) may yield free epitopes blocking otherwise cytotoxic antibodies. Such a scenario may be relevant for the outcome of *G. lamblia* infections because cytotoxic anti-VSP antibodies turned out to possess a modulatory function on the proliferative parasite population characteristics in the experimental murine host as reviewed elsewhere (Müller and Gottstein, 1998; Müller and von Allmen, 2005). Moreover, VSPs may have enzymatic functions as evidenced in the case of VSP9B10A (ORF 101074; VSP88), which has cysteine protease activity (Cabrera-Licona *et al*., 2017). Other functions such as enzyme activities or interactions with receptors on other trophozoites or on host cells remain to be elucidated.

Since the WB isolate issues from a patient treated with MET, the clones A1 and C6 may represent two different strategies to deal with this drug pressure. WBC6 retains a higher flexibility with respect to gene expression and is able to generate a highly nitro drug-resistant offspring in a few selection cycles (Müller *et al*., 2007) differing from the original clone with respect to appropriate physiological adaptations and an adapted proteome (Müller *et al*., 2018; Müller *et al*., 2019). Conversely, WBA1 may represent a ‘freezed’ status with a moderate nitro drug resistance at the price of a loss in gene expression flexibility.

This hypothesis including the identification of elements controlling this flexibility, e.g. in the differential signalome of both strains as evidenced in our dataset, would require further investigations.

## Supplementary material

For supplementary material accompanying this paper visit https://doi.org/10.1017/S0031182020000657. click here to view supplementary material

## References

[ref1] Adam RD (2001) Biology of *Giardia lamblia*. Clinical Microbiology Reviews 14, 447–475. 11432808 10.1128/CMR.14.3.447-475.2001 PMC88984

[ref2] Adam RD, Aggarwal A, Lal AA, de La Cruz VF, McCutchan T and Nash TE (1988) Antigenic variation of a cysteine-rich protein in *Giardia lamblia*. Journal of Experimental Medicine 167, 109–118. 3335828 10.1084/jem.167.1.109 PMC2188815

[ref3] Adam RD, Nigam A, Seshadri V, Martens CA, Farneth GA, Morrison HG, Nash TE, Porcella SF and Patel R (2010) The *Giardia Lamblia* vsp gene repertoire: characteristics, genomic organization, and evolution. BMC Genomics 11, 424. 20618957 10.1186/1471-2164-11-424 PMC2996952

[ref4] Allain T, Amat CB, Motta JP, Manko A and Buret AG (2017) Interactions of *Giardia* sp. with the intestinal barrier: epithelium, mucus, and microbiota. Tissue Barriers 5, e1274354. 28452685 10.1080/21688370.2016.1274354 PMC5362998

[ref5] Bénéré E, da Luz RA, Vermeersch M, Cos P and Maes L (2007) A new quantitative in vitro microculture method for *Giardia Duodenalis* trophozoites. Journal of Microbiological Methods 71, 101–106. 17888535 10.1016/j.mimet.2007.07.014

[ref6] Bienz M, Siles-Lucas M, Wittwer P and Müller N (2001) vsp gene expression by *Giardia lamblia* clone GS/M-83-H7 during antigenic variation *in vivo* and *in vitro*. Infection and Immunity 69, 5278–5285. 11500396 10.1128/IAI.69.9.5278-5285.2001 PMC98636

[ref7] Bradford MM (1976) A rapid and sensitive method for the quantitation of microgram quantities of protein utilizing the principle of protein-dye binding. Analytical Biochemistry 72, 248–254. 942051 10.1016/0003-2697(76)90527-3

[ref8] Brauner A, Fridman O, Gefen O and Balaban NQ (2016) Distinguishing between resistance, tolerance and persistence to antibiotic treatment. Nature Reviews Microbiology 14, 320–330. 27080241 10.1038/nrmicro.2016.34

[ref9] Cabrera-Licona A, Solano-Gonzalez E, Fonseca-Linan R, Bazan-Tejeda ML, Raul A-G, Bermudez-Cruz RM and Ortega-Pierres G (2017) Expression and secretion of the *Giardia duodenalis* variant surface protein 9B10A by transfected trophozoites causes damage to epithelial cell monolayers mediated by protease activity. Experimental Parasitology 179, 49–64. 28668253 10.1016/j.exppara.2017.06.006

[ref10] Campbell JD and Faubert GM (1994) Comparative studies on *Giardia lamblia* encystation in vitro and in vivo. Journal of Parasitology 80, 36–44. 8308656

[ref11] Carranza PG, Feltes G, Ropolo A, Quintana SM, Touz MC and Luján HD (2002) Simultaneous expression of different variant-specific surface proteins in single *Giardia lamblia* trophozoites during encystation. Infection and Immunity 70, 5265–5268. 12183579 10.1128/IAI.70.9.5265-5268.2002 PMC128263

[ref12] Cernikova L, Faso C and Hehl AB (2018) Five facts about *Giardia lamblia*. PLoS Pathogens 14, e1007250. 30261050 10.1371/journal.ppat.1007250 PMC6160191

[ref13] Dou M, Clair G, Tsai CF, Xu K, Chrisler WB, Sontag RL, Zhao R, Moore RJ, Liu T, Pasa-Tolic L, Smith RD, Shi T, Adkins JN, Qian WJ, Kelly RT, Ansong C and Zhu Y (2019) High-throughput single cell proteomics enabled by multiplex isobaric labeling in a nanodroplet sample preparation platform. Analytical Chemistry 91, 13119–13127. 31509397 10.1021/acs.analchem.9b03349 PMC7192326

[ref14] Emery-Corbin SJ, Vuong D, Lacey E, Svard SG, Ansell BRE and Jex AR (2018) Proteomic diversity in a prevalent human-infective *Giardia duodenalis* sub-species. International Journal for Parasitology 48, 817–823. 30059689 10.1016/j.ijpara.2018.05.003

[ref15] Emery SJ, van Sluyter S and Haynes PA (2014) Proteomic analysis in *Giardia duodenalis* yields insights into strain virulence and antigenic variation. Proteomics 14, 2523–2534. 25266764 10.1002/pmic.201400144

[ref16] Emery SJ, Lacey E and Haynes PA (2015) Quantitative proteomic analysis of *Giardia duodenalis* assemblage A: a baseline for host, assemblage, and isolate variation. Proteomics 15, 2281–2285. 25728068 10.1002/pmic.201400434

[ref17] Emery SJ, Baker L, Ansell BRE, Mirzaei M, Haynes PA, McConville MJ, Svard SG and Jex AR (2018) Differential protein expression and post-translational modifications in metronidazole-resistant *Giardia duodenalis*. Gigascience 7. doi:10.1093/gigascience/giy024. PMC5913674 29688452

[ref18] Faso C and Hehl AB (2011) Membrane trafficking and organelle biogenesis in *Giardia lamblia*: use it or lose it. International Journal for Parasitology 41, 471–480. 21296082 10.1016/j.ijpara.2010.12.014

[ref19] Furfine ES and Wang CC (1990) Transfection of the *Giardia lamblia* double-stranded RNA virus into giardia lamblia by electroporation of a single-stranded RNA copy of the viral genome. Molecular and Cellular Biology 10, 3659–3662. 2355918 10.1128/mcb.10.7.3659-3662.1990 PMC360806

[ref20] Gardner TB and Hill DR (2001) Treatment of giardiasis. Clinical Microbiology Reviews 14, 114–128. 11148005 10.1128/CMR.14.1.114-128.2001 PMC88965

[ref21] Gillin FD, Hagblom P, Harwood J, Aley SB, Reiner DS, McCaffery M, So M and Guiney DG (1990) Isolation and expression of the gene for a major surface protein of *Giardia lamblia*. Proceedings of the National Academy of Sciences of the USA 87, 4463–4467. 2352929 10.1073/pnas.87.12.4463 PMC54135

[ref22] Gottstein B, Harriman GR, Conrad JT and Nash TE (1990) Antigenic variation in *Giardia lamblia*: cellular and humoral immune response in a mouse model. Parasite Immunology 12, 659–673. 1707508 10.1111/j.1365-3024.1990.tb00995.x

[ref23] Hemphill A, Müller N and Müller J (2019) Comparative pathobiology of the intestinal protozoan parasites *Giardia lamblia*, *Entamoeba histolytica*, and *Cryptosporidium parvum*. Pathogens (Basel, Switzerland) 8. doi: 10.3390/pathogens8030116. PMC6789772 31362451

[ref24] Heyworth MF (2016) *Giardia duodenalis* genetic assemblages and hosts. Parasite 23, 13. 26984116 10.1051/parasite/2016013 PMC4794627

[ref25] Kulakova L, Singer SM, Conrad J and Nash TE (2006) Epigenetic mechanisms are involved in the control of *Giardia lamblia* antigenic variation. Molecular Microbiology 61, 1533–1542. 16968226 10.1111/j.1365-2958.2006.05345.x

[ref26] Litleskare S, Rortveit G, Eide GE, Hanevik K, Langeland N and Wensaas KA (2018) Prevalence of irritable bowel syndrome and chronic fatigue 10 years after *Giardia* infection. Clinical Gastroenterology and Hepatology 16, 1064–1072 e1064. 29378314 10.1016/j.cgh.2018.01.022

[ref27] Liu D, Paczkowski P, Mackay S, Ng C and Zhou J (2020) Single-cell multiplexed proteomics on the IsoLight resolves cellular functional heterogeneity to reveal clinical responses of cancer patients to immunotherapies. Methods in Molecular Biology 2055, 413–431. 31502163 10.1007/978-1-4939-9773-2_19

[ref28] Meng TC, Hetsko ML and Gillin FD (1993) Antigenic switching of TSA 417, a trophozoite variable surface protein, following completion of the life cycle of *Giardia lamblia*. Infection and Immunity 61, 5394–5397. 8225614 10.1128/iai.61.12.5394-5397.1993 PMC281329

[ref29] Morrison HG, McArthur AG, Gillin FD, Aley SB, Adam RD, Olsen GJ, Best AA, Cande WZ, Chen F, Cipriano MJ, Davids BJ, Dawson SC, Elmendorf HG, Hehl AB, Holder ME, Huse SM, Kim UU, Lasek-Nesselquist E, Manning G, Nigam A, Nixon JE, Palm D, Passamaneck NE, Prabhu A, Reich CI, Reiner DS, Samuelson J, Svard SG and Sogin ML (2007) Genomic minimalism in the early diverging intestinal parasite *Giardia lamblia*. Science (New York, N.Y.) 317, 1921–1926. 17901334 10.1126/science.1143837

[ref30] Mowatt MR, Nguyen BY, Conrad JT, Adam RD and Nash TE (1994) Size heterogeneity among antigenically related *Giardia lamblia* variant-specific surface proteins is due to differences in tandem repeat copy number. Infection and Immunity 62, 1213–1218. 7510666 10.1128/iai.62.4.1213-1218.1994 PMC186261

[ref31] Müller N and Gottstein B (1998) Antigenic variation and the murine immune response to *Giardia lamblia*. International Journal for Parasitology 28, 1829–1839. 9925261 10.1016/s0020-7519(98)00137-4

[ref32] Müller J and Hemphill A (2013) New approaches for the identification of drug targets in protozoan parasites. International Review of Cell and Molecular Biology 301, 359–401. 23317822 10.1016/B978-0-12-407704-1.00007-5

[ref33] Müller N and von Allmen N (2005) Recent insights into the mucosal reactions associated with *Giardia lamblia* infections. International Journal for Parasitology 35, 1339–1347. 16182298 10.1016/j.ijpara.2005.07.008

[ref34] Müller N, Stäger S and Gottstein B (1996) Serological analysis of antigenic heterogeneity of *Giardia lamblia* variant surface proteins. Infection and Immunity 64, 1385–1390. 8606105 10.1128/iai.64.4.1385-1390.1996 PMC173930

[ref35] Müller J, Sterk M, Hemphill A and Müller N (2007) Characterization of *Giardia lamblia* WB C6 clones resistant to nitazoxanide and to metronidazole. Journal of Antimicrobial Chemotherapy 60, 280–287. 17561498 10.1093/jac/dkm205

[ref36] Müller J, Rout S, Leitsch D, Vaithilingam J, Hehl A and Müller N (2015) Comparative characterisation of two nitroreductases from *Giardia lamblia* as potential activators of nitro compounds. International Journal for Parasitology. Drugs and Drug Resistance 5, 37–43. 27099829 10.1016/j.ijpddr.2015.03.001 PMC4813764

[ref37] Müller J, Hemphill A and Müller N (2018) Physiological aspects of nitro drug resistance in *Giardia lamblia*. International Journal for Parasitology. Drugs and Drug Resistance 8, 271–277. 29738984 10.1016/j.ijpddr.2018.04.008 PMC6039359

[ref38] Müller J, Braga S, Heller M and Müller N (2019) Resistance formation to nitro drugs in *Giardia lamblia*: no common markers identified by comparative proteomics. International Journal for Parasitology: Drugs and Drug Resistance 9, 112–119. 30889439 10.1016/j.ijpddr.2019.03.002 PMC6423486

[ref39] Nash TE (2001) Treatment of *Giardia lamblia* infections. Pediatric Infectious Disease Journal 20, 193–195. 11224841 10.1097/00006454-200102000-00015

[ref40] Nash TE (2002) Surface antigenic variation in *Giardia lamblia*. Molecular Microbiology 45, 585–590. 12139606 10.1046/j.1365-2958.2002.03029.x

[ref41] Nash TE and Mowatt MR (1992) Characterization of a *Giardia lamblia* variant-specific surface protein (VSP) gene from isolate GS/M and estimation of the VSP gene repertoire size. Molecular and Biochemical Parasitology 51, 219–227. 1574080 10.1016/0166-6851(92)90072-r

[ref42] Nash TE, McCutchan T, Keister D, Dame JB, Conrad JD and Gillin FD (1985) Restriction-endonuclease analysis of DNA from 15 *Giardia* isolates obtained from humans and animals. Journal of Infectious Diseases 152, 64–73. 2409186 10.1093/infdis/152.1.64

[ref43] Nash TE, Aggarwal A, Adam RD, Conrad JT and Merritt JW (1988) Antigenic variation in *Giardia lamblia*. Journal of Immunology 141, 636–641. 2454999

[ref44] Nash TE, Banks SM, Alling DW, Merritt JW and Conrad JT (1990 *a*) Frequency of variant antigens in *Giardia lamblia*. Experimental Parasitology 71, 415–421. 1699782 10.1016/0014-4894(90)90067-m

[ref45] Nash TE, Conrad JT and Merritt JW (1990 *b*) Variant specific epitopes of *Giardia lamblia*. Molecular and Biochemical Parasitology 42, 125–132. 1700296 10.1016/0166-6851(90)90120-b

[ref46] Nash TE, Herrington DA, Levine MM, Conrad JT and Merritt JW (1990 *c*) Antigenic variation of *Giardia lamblia* in experimental human infections. Journal of Immunology 144, 4362–4369. 2341723

[ref47] Nash TE, Luján HT, Mowatt MR and Conrad JT (2001) Variant-specific surface protein switching in *Giardia lamblia*. Infection and Immunity 69, 1922–1923. 11179375 10.1128/IAI.69.3.1922-1923.2001 PMC98104

[ref48] Perez-Riverol Y, Csordas A, Bai J, Bernal-Llinares M, Hewapathirana S, Kundu DJ, Inuganti A, Griss J, Mayer G, Eisenacher M, Perez E, Uszkoreit J, Pfeuffer J, Sachsenberg T, Yilmaz S, Tiwary S, Cox J, Audain E, Walzer M, Jarnuczak AF, Ternent T, Brazma A and Vizcaino JA (2019) The PRIDE database and related tools and resources in 2019: improving support for quantification data. Nucleic Acids Research 47, D442–D450. 30395289 10.1093/nar/gky1106 PMC6323896

[ref49] Prochaska HJ and Santamaria AB (1988) Direct measurement of NAD(P)H:quinone reductase from cells cultured in microtiter wells: a screening assay for anticarcinogenic enzyme inducers. Analytical Biochemistry 169, 328–336. 3382006 10.1016/0003-2697(88)90292-8

[ref50] Prucca CG and Lujan HD (2009) Antigenic variation in *Giardia lamblia*. Cellular Microbiology 11, 1706–1715. 19709056 10.1111/j.1462-5822.2009.01367.x

[ref51] Prucca CG, Slavin I, Quiroga R, Elías EV, Rivero FD, Saura A, Carranza PG and Luján HD (2008) Antigenic variation in *Giardia lamblia* is regulated by RNA interference. Nature 456, 750–754. 19079052 10.1038/nature07585

[ref52] Prucca CG, Rivero FD and Luján HD (2011) Regulation of antigenic variation in *Giardia lamblia*. Annual Review of Microbiology 65, 611–630. 10.1146/annurev-micro-090110-102940 21740226

[ref53] Smith PD, Gillin FD, Kaushal NA and Nash TE (1982 *a*) Antigenic analysis of *Giardia lamblia* from Afghanistan, Puerto Rico, Ecuador, and Oregon. Infection and Immunity 36, 714–719. 7085076 10.1128/iai.36.2.714-719.1982 PMC351289

[ref54] Smith PD, Gillin FD, Spira WM and Nash TE (1982 *b*) Chronic giardiasis: studies on drug sensitivity, toxin production, and host immune response. Gastroenterology 83, 797–803. 7106510

[ref55] Sun CH, Chou CF and Tai JH (1998) Stable DNA transfection of the primitive protozoan pathogen *Giardia lamblia*. Molecular and Biochemical Parasitology 92, 123–132. 9574916 10.1016/s0166-6851(97)00239-9

[ref56] Svärd SG, Meng TC, Hetsko ML, McCaffery JM and Gillin FD (1998) Differentiation-associated surface antigen variation in the ancient eukaryote *Giardia lamblia*. Molecular Microbiology 30, 979–989. 9988475 10.1046/j.1365-2958.1998.01125.x

[ref57] Thompson RC (2004) The zoonotic significance and molecular epidemiology of *Giardia* and giardiasis. Veterinary Parasitology 126, 15–35. 15567577 10.1016/j.vetpar.2004.09.008

[ref58] Wagner BD (2009) The use of coumarins as environmentally-sensitive fluorescent probes of heterogeneous inclusion systems. Molecules 14, 210–237. 19127249 10.3390/molecules14010210 PMC6253935

[ref59] Xu F, Jex A and Svard SG (2020) A chromosome-scale reference genome for *Giardia intestinalis* WB. Scientific Data 7, 38. 32019935 10.1038/s41597-020-0377-y PMC7000408

[ref60] Yang YM and Adam RD (1995) Analysis of a repeat-containing family of *Giardia lamblia* variant-specific surface protein genes: diversity through gene duplication and divergence. Journal of Eukaryotic Microbiology 42, 439–444. 7581319 10.1111/j.1550-7408.1995.tb05888.x

[ref61] Zahedi A, Field D and Ryan U (2017) Molecular typing of *Giardia duodenalis* in humans in Queensland – first report of assemblage E. Parasitology 144, 1154–1161. 28482937 10.1017/S0031182017000439

[ref62] Zamponi N, Zamponi E, Mayol GF, Lanfredi-Rangel A, Svard SG and Touz MC (2017) Endoplasmic reticulum is the sorting core facility in the Golgi-lacking protozoan *Giardia lamblia*. Traffic (Copenhagen, Denmark) 18, 604–621. 28696565 10.1111/tra.12501

