# Supplementary material for: Comparative proteomics of three Giardia lamblia strains: investigation of antigenic variation in the post-genomic era
Source: Parasitology. 2020 Apr 27;147(9):1008–18. doi: 10.1017/S0031182020000657 (PMC7332775; doi:10.1017/S0031182020000657)
Supplement: Supplementary file 1 [file S0031182020000657sup.zip › S0031182020000657sup003.docx]

**Supplemental information**

**Table S1. Proteome data set from** *G. lamblia* **trophozoites, strains WBC6, WBA1, and GS/M-H7.** Trophozoites of the resistant strains were grown and subjected to MS shotgun analysis as described in Materials and Methods. [See Excel spreadsheet PAR_2020_0020_assemblage_A_B_Full_dataset_PRIDE]

**Table S2. Single peptide dataset set from** *G. lamblia* **trophozoites, strains WBC6, WBA1, and GS/M-H7.** Trophozoites of the resistant strains were grown and subjected to MS shotgun analysis as described in Materials and Methods. [See Excel spreadsheet PAR_2020_0020_assemblage_A_B_peptides]

**Table S3. Major surface antigens** **expressed in** *G. lamblia* **WBC6 trophozoites grown on two different media.** Trophozoites of the resistant strains were either grown as described in Materials and Methods (this work; referred to as “old medium”), or on a culture medium containing heat-inactivated fetal bovine serum (Biochrome, Berlin, Germany) instead of adult bovine serum (Biofluids, Rockville MD) and casein peptone from a different provider (Merck, Darmstadt, Germany instead of Becton Dickinson, Cockeysville, MD; referred to as “new medium”). The complete dataset corresponding to the trophozoites grown on “new medium” has been published elsewhere. Mean values ± standard deviations of LFQ values (x10^6^) are given for three biological replicates. Nd, not detected. VSPs with mean LFQ levels > 10^8^ are highlighted.

| **Accession N°** | **Old annotation** | **“old medium”** | **“new medium”** |
| --- | --- | --- | --- |
| GL50803_137740 | VSP3 | 5.9 ± 0.3 | 45.0 ± 13.8 |
| GL50803_101074 | VSP88 | **427.3 ± 16.8** | **185.5 ± 7.0** |
| GL50803_112801 | VSP169 | 2.6 ± 0.8 | nd |
| GL50803_113450 | VSP44 (VSP417-4) | **273.3 ± 11.0** | 44.5 ± 10.8 |
| GL50803_113797 | VSP5 (TSA417 partial) | **3847.3 ± 86.0** | 11.0 ± 2.1 |
| GL50803_116477 | VSP149 | **491.4 ± 15.0** | nd |
| GL50803_13194 | VSP38 | 18.5 ± 2.1 | 30.1 ± 3.1 |
| GL50803_137608 | VSP175 | 1.0 ± 0.2 | nd |
| GL50803_137612 | VSP160 | **152.4 ± 4.2** | **147.1 ± 8.5** |
| GL50803_137613 | VSP188 | **835.6 ± 39.2** | **702.7 ± 46.1** |
| GL50803_137617 | VSP77 | 79.3 ± 0.2 | **107.0 ± 4.4** |
| GL50803_137618 | VSP8 | **1998.4 ± 119.5** | **1027.6 ± 70.9** |
| GL50803_14586 | VSP186 | **437.9 ± 9.2** | 97.1 ± 2.0 |
| GL50803_221693 | VSPA6 (CRP170) | 5.0 ± 3.6 | **646.9 ± 31.1** |
| GL50803_33279 | VSP100 | 49.9 ± 2.1 | 29.7 ± 3.4 |
| GL50803_41472 | VSP49 | 41.4 ± 1.9 | 7.7 ± 1.9 |
| GL50803_5812 | VSP121 | **107.6 ± 20.6** | nd |

**Figure S1. CRP170 aligns to the hypothetical protein GL50803_221693.** The CRP170 partial) sequence (P15799.1; red bar in Fig. S1 C) was BLASTed against non-redundant proteins. A, maximal and total scores of the three proteins sharing E value 0.0, query cover and identity 100% with CRP170. B, total scores of closest homologues (E= 0.0 for all proteins) found in the GiardiaDB to the three proteins listed in A. C, graphical alignment of the four sequences. VSP, variant-specific surface protein.


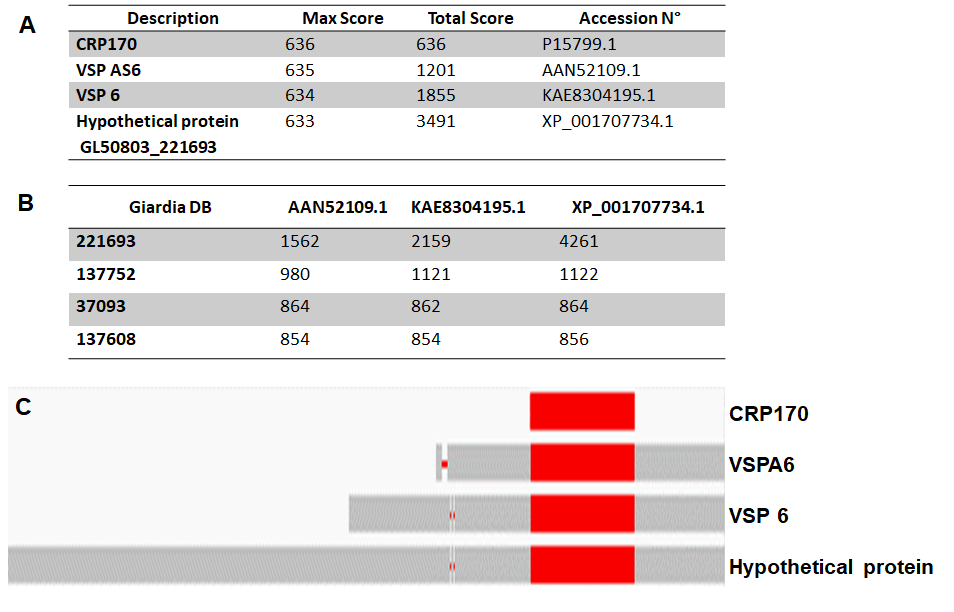


| **Figure S2. Mapping of identified peptides to the coding sequence of GL50803_221693.** The identified sequences are highlighted in bold red letters. The highly repetitive region TNPSDPTGTCVSAVDCQGSAGYYTDDSVSDAKECKKCNA  PCTACAGTADKCTKCDANGAAPYLKK is highlighted in yellow. The GAAPYLKK motif recognized by mAb6E7 printed in blue. | **AA #** |
| --- | --- |
| MCPRFRWPLCSIEFRDMKQDPVRLLMQTRAVLCQLLASMLVGIGLIFAVALADACSPSIDGCAECDSTGKKCTK**CDANGNTPYLKKTNPSDPTGTCVSAVDCQGSAGYYTDDSVSDAKECKKCNAPCTACAGTADKCTKCDANGAAPYLKK**TNPSDPTGTCVSAVDCQGSAGYYTDDSVSDAKECKKCNAPCTACAGTADKCTKCDANGAAPYLKKTNPSDPTGTCVSAVDCQGSAGYYTDDSVSDAKECKKCNAPCTACAGTADKCTKCDANGAAPYLKKTNPSDPTGTCVSAVDCQGSAGYYTDDSVSDAKECKKCNAPCTACAGTADKCTKCDANGAAPYLKKTNPSDPTGTCVSAVDCQGSAGYYTDDSVSDAKECKKCNAPCTACAGTADKCTKCDANGAAPYLKKTNPSDPTGTCVSAVDCQGSAGYYTDDSVSDAKECKKCNAPCTACAGTADKCTKCDANGAAPYLKKTNPSDPTGTCVSAVDCQGSAGYYTDDSVSDAKECKKCNAPCTACAGTADKCTKCDANGAAPYLKKTNPSDPTGTCVSAVDCQGSAGYYTDDSVSDAKECKKCNAPCTACAGTADKCTKCDANGAAPYLKKTNPSDPTGTCVSAVDCQGSAGYYTDDSVSDAKECKKCNAPCTACAGTADKCTKCDANGAAPYLKKTNPSDPTGTCVSAVDCQGSAGYYTDDSVSDAKECKKCNAPCTACAGTADKCTKCDANGAAPYLKKTNPSDPTGTCVSAVDCQGSAGYYTDDSVSDAKECKKCNAPCTACAGTADKCTKCDANGAAPYLKKTNPSDPTGTCVSAVDCQGSAGYYTDDSVSDAKECKKCNAPCTACAGTADKCTKCDANGAAPYLKKTNPSDPTGTCVSAVDCQGSAGYYTDDSVSDAKECKKCNAPCTACAGTADKCTKCDANGAAPYLKKTNPSDPTGTCVSAVDCQGSAGYYTDDSVSDAKECKKCNAPCTACAGTADKCTKCDANGAAPYLKKTNPSDPTGTCVSAVDCQGSAGYYTDDSVSDAKECKKCNAPCTACAGTADKCTKCDANGAAPYLKKTNPSDPTGTCVSAVDCQGSAGYYTDDSVSDAKECKKCNAPCTACAGTADKCTKCDANGAAPYLKKTNPSDPTGTCVSAVDCQGSAGYYTDDSVSDAKECKKCNAPCTACAGTADKCTKCDANGAAPYLKKTNPSDPTGTCVSAVDCQGSAGYYTDDSVSDAKECKKCNAPCTACAGTADKCTKCDANGAAPYLKKTNPSDPTGTCVSAVDCQGSAGYYTDDSVSDAKECKKCNAPCTACAGTADKCTKCDANGAAPYLKKTNPSDPTGTCVSAVDCQGSAGYYTDDSVSDAKECKKCNAPCTACAGTADKCTKCDANGAAPYLKKTNPSDPTGTCVSAVDCQGSAGYYTDDSVSDAKECKKCNAPCTACAGTADKCTKCDANGAAPYLKKTNPSDPTGTCVSAVDCQGSAGYYTDDSVSDAKECKKCNAPCTACAGTADKCTKCDANGAAPYLKKTNPSDPTGTCVSAVDCQGSAGYYTDDSVSDAKECKKCNAPCTACAGTADKCTKCDANGAAPYLKKTNPSDPTGTCVSAVDCQGSAGYYTDDSVSDAKECKKCNAPCTACAGTADKCTKCDANGAAPYLKKTNPSDPTGTCVSAVDCQGSAGYYTDDSVSDAKECKKCNAPCTACAGTADKCTKCDANGAAPYLKKTNPSDPTGTCVSAVDCQGSAGYYTDDSVSDAKECKKCNAPCTACAGTADKCTKCDANGAAPYLKKTNPSDPTGTCVSAVDCQGSAGYYTDDSVSDAKECK**KCAEGQKPNTAGTQCFSCSDANCERCDQNDVCARCSTGAPPENGK**CPAATPGCHSSCDGCTENAMTNQADKCTGCKEGRYLKPESAAGQSGACLTAEECTSDKTHFTREKAGDSK**GMCLSCSDATHGITGCKK**CALK**TLSGEAESTVVCSECTDKR**LTPSGNACLDNCPAGTYADNINGVSVCASCHATCAECNGDANAASCTACYPGYSLLYGSGTAGTCVK**ECTGAFITNCADGQCTANVGGAKYCAQCKDGYAPIDGICTTVAAAGRDASVCTAADGK**CTK**CAGEYTLMSGGCYGVAK**LPGK**SVCTLASNGKCTMCAANNQAPVQEKCPECSEGCAKCNDSNACTECLPGYYKGAGDK**CFK**CTASSGNNNQITGVANCVSCAPPAGGNGGPVTCYIKTDGDNTGGSVNK**SGLSTGAIAGISVAVVVVVGGLVGFLCWWFICRGKA | 60  120  180  240  300  360  420  480  540  600  660  720  780  840  900  960  1020  1080  1140  1200  1260  1320  1380  1440  1500  1560  1620  1680  1740  1800  1860  1920  1980  2040  2100  2160  2220  2259 |
